# Recent neuroimaging, neurophysiological, and neuropathological advances for the understanding of NPC

**DOI:** 10.12688/f1000research.12361.1

**Published:** 2018-02-15

**Authors:** Alberto Benussi, Maria Sofia Cotelli, Alessandro Padovani, Barbara Borroni

**Affiliations:** 1 Neurology Unit, Department of Clinical and Experimental Sciences, University of Brescia, Viale Europa, 11, 25123 Brescia BS, Italy; 2 Neurology Unit, Valle Camonica Hospital, 25040 Esine, Brescia, Italy

**Keywords:** Niemann–Pick disease, Miglustat, neuroimaging, neurophysiological, neuropathological

## Abstract

Niemann–Pick disease type C (NPC) is a rare autosomal recessive lysosomal storage disorder with extensive biological, molecular, and clinical heterogeneity. Recently, numerous studies have tried to shed light on the pathophysiology of the disease, highlighting possible disease pathways common to other neurodegenerative disorders, such as Alzheimer’s disease and frontotemporal dementia, and identifying possible candidate biomarkers for disease staging and response to treatment. Miglustat, which reversibly inhibits glycosphingolipid synthesis, has been licensed in the European Union and elsewhere for the treatment of NPC in both children and adults. A number of ongoing clinical trials might hold promise for the development of new treatments for NPC. The objective of the present work is to review and evaluate recent literature data in order to highlight the latest neuroimaging, neurophysiological, and neuropathological advances for the understanding of NPC pathophysiology. Furthermore, ongoing developments in disease-modifying treatments will be briefly discussed.

## Introduction

Niemann–Pick disease type C (NPC) (MIM 257220) is an autosomal recessive neurovisceral lysosomal lipid storage disorder due to mutations of either the *NPC1* (95% of families) ^1^ or the *NPC2* gene ^2^. The incidence of NPC is estimated to be 1 in 100,000 live births ^3– 5^, although the late-onset phenotypes or variant forms with visceral-only or neurologically mild presentations might have a much higher frequency ^6^.

Although the exact functions of the *NPC1* and *NPC2* genes are still to be fully elucidated, mutations in these genes cause a loss of function, resulting in the accumulation of unesterified cholesterol and glycosphingolipids within the late endosome/lysosome of all cells, leading to downstream effects on cholesterol homeostasis ^7– 9^. Recently, using structural biology approaches, such as crystallography and cryo–electron microscopy, researchers have gained insights into how NPC1 and NPC2 proteins function in tandem to facilitate cholesterol efflux from late endosomes/lysosomes ^10– 19^.

The broad clinical spectrum ranges from a neonatal, rapidly fatal disease to an adult-onset chronic neurodegenerative disorder, and patients can be classified into four general categories based on age at neurological onset: the early infantile, late infantile, juvenile, and adolescent/adult onset form ^3^. In most patients, disease severity is defined by the neurological involvement but usually is preceded by systemic signs such as neonatal cholestatic jaundice or isolated spleen or liver enlargement in infancy or childhood ^4^. In the majority of patients with NPC, the liver disease frequently resolves with time ^3, 4^.

Clinical pictures of NPC are widely heterogeneous, and there are a number of neurological signs and symptoms with different ages at onset and different rates of progression: gait ataxia, clumsiness, cataplexy, epilepsy, dystonia, supranuclear gaze palsy, dysarthria, dysphagia, cerebellar ataxia, psychiatric illnesses, or cognitive decline ^3, 4, 20^. Psychiatric disturbances may be undiagnosed for several years and are often characterized by psychosis such as paranoid delusions, visual or auditory hallucinations, or behavioral abnormalities ^20^. The age of onset of neurological manifestations usually correlates with the patient’s prognosis ^4^.

Slightly different phenotypes have been observed in patients with *NPC2* gene mutations, which are frequently associated with a severe phenotype, characterized by pulmonary infiltrates, respiratory failure, and death in early age ^21– 23^. Adult-onset disease with frontal lobe atrophy has also been described ^24^, and in some cases prolonged survival into middle adult life has been reported ^25^.

Various NPC disability scales have been developed ^26– 30^; however, the original scale by Iturriaga, ^29^ and the subsequent modified versions are the most widely adopted ^27, 28^.

The diagnostic workup currently includes a combination of both biochemical and genetic analysis. Plasma cholesterol oxidation products (oxysterols), including cholestane-3β,5α,6β-triol ^31, 32^ and filipin staining in cultured fibroblasts ^33– 35^ are considered key in the diagnostic workup.

Plasma oxysterols have been shown to be highly sensitive for NPC; however, the originally reported specificity has recently been widened to other disorders of sterol metabolism, including acid sphingomyelinase deficiency (Niemann–Pick type A and B disease), cerebrotendinous xanthomatosis, and lysosomal acid lipase deficiency (Wolman disease) ^36– 38^. Furthermore, oxysterols can be increased in neonates with non-NPC cholestasis ^39^.

Until recently, filipin staining was considered the gold standard assay for NPC diagnosis ^4, 9, 33, 40^, the typical pattern was observed in 80 to 85% of cases with NPC ^33, 41– 43^, and a positive staining occurred in 80 to 100% of cells ^33^. Only one condition, mucolipidosis II/III, is known to resemble a classic NPC filipin profile ^33^. In the remaining 15 to 20% of NPC cases, a “variant” profile is observed, and only 50 to 80% cells stain positive ^33^. A “variant” profile can also be observed in various conditions, including NPC heterozygous carriers ^44^, Niemann–Pick type A and B disease ^45^, MEGDEL (3-methylglutaconic aciduria, deafness, encephalopathy, and Leigh-like disease) syndrome ^46^, Smith-Lemli-Opitz syndrome ^47^, and Tangier disease ^48^.

A major limitation of the filipin test is that it requires cell cultures of living fibroblasts from a skin biopsy, has relatively long turnaround times, and is performed in only a few specialized laboratories worldwide ^49^.

Recently, a bile acid–based newborn screening for NPC, which identified 3β,5α,6β-trihydroxycholanic acid and its glycine conjugate, metabolites of cholestane-3β,5α,6β-triol, on dried blood spots, provided 100% specificity and sensitivity in identifying patients with NPC ^50^.

Genetic testing, which involves the sequencing of the *NPC1* and *NPC2* genes, is also available; however, it is still inconclusive in 12 to 15% cases because of the unknown pathogenicity of the mutation, the lack of a study of allele segregation, and the possible existence of one unidentified mutant allele ^6^.

A series of therapies for NPC are actively being developed. Miglustat, an iminosugar that is a competitive inhibitor of glucosylceramide synthase and specifically targets the metabolic pathway that leads to the synthesis of glycosphingolipids in neurons and other cells, has been approved for the treatment of NPC in Europe and elsewhere ^51^.

Very recently, the most up-to-date clinical guidelines on NPC were published by an expert group ^52^.

In recent years, numerous studies have increased the understanding of NPC, highlighting the very complex and multifaceted nature of the disease, and reported possible links to other neurodegenerative disorders, such as Alzheimer’s disease (AD) and frontotemporal dementia (FTD).

The objective of the present work is to review and evaluate recent literature data in order to highlight the latest neuroimaging, neurophysiological, and neuropathological advances for the understanding of NPC pathophysiology. Furthermore, recent developments in disease-modifying treatments will be briefly discussed.

## Neuroimaging

Historically, brain magnetic resonance imaging (MRI) and computed tomography scans were not usually considered very useful for the diagnosis of NPC, as most of the findings are unspecific, sometimes showing cerebellar or cortical atrophy or, in the severe infantile form, white matter changes ^3^. However, in recent years, numerous studies have shown the involvement of several cerebral structures in patients with NPC, even highlighting the possible modifications induced by therapeutic interventions ( Table 1).

**Table 1.** Proposed diagnostic and prognostic markers for Niemann–Pick disease type C.

Variable	Marker	References
Brain MRI-VBM	Hippocampus, thalamus, striatum, cerebellum atrophy	53– 57
Brain MRI	Pontine-to-midbrain ratio atrophy	59
Brain MRI-DTI	Corpus callosum, corona radiate, cingulate gyrus decreased fractional anisotropy	61– 64
Brain H-MRSI	Frontal and parietal cortices, centrum semiovale, caudate nucleus decreased N-acetyl aspartate/ Creatine ratio	73, 74
^123^I-FP-CIT (Ioflupane I 123 DaTScan)	Symmetrical loss of dopamine transporter binding	57– 60
Brain FDG-PET	Frontal and temporal lobe hypometabolism	68, 72
Brain SPECT	Frontal and temporal lobe hypoperfusion	69
TMS	SAI and LTP-like cortical plasticity impairment	60, 93

DTI, diffusion tensor imaging; FDG-PET, ^18^F-fluorodeoxyglucose positron emission tomography; H-MRSI, proton magnetic resonance spectroscopic imaging; MRI-VBM, magnetic resonance imaging–voxel-based morphometry; SAI, short-latency afferent inhibition; SPECT, single-photon emission computed tomography; TMS, transcranial magnetic stimulation.

Voxel-based morphometry analyses have shown a significant involvement of the hippocampus, thalamus, striatum, and cerebellum in NPC ^53– 57^. In particular, cerebellar gray matter and left thalamus volume loss were significantly correlated with Iturriaga disability scale changes and ataxia measures ^53, 58^. Furthermore, untreated patients exhibited what may appear to be greater thalamic and cerebellar gray and white matter reductions over time compared with both controls and patients treated with miglustat ^53^.

As in progressive supranuclear palsy, the pontine-to-midbrain ratio is increased in adult patients with NPC compared with controls, and the strong correlation with illness and oculomotor variables suggests that it may be a useful marker for illness progression in NPC ^59^.

The atrophy pattern in the thalamus, hippocampus, and caudate nucleus, observed with cortical thickness analyses, showed a significant correlation with memory, executive functions, and motor control dysfunction ^58^.

The involvement of deep gray nuclei has also been confirmed by ^123^I-FP-CIT (ioflupane I 123 DaTSCAN) single-photon emission computed tomography (SPECT) imaging in a case of NPC, showing a marked, symmetrical loss of dopamine transporter binding, especially in the putamen ^57^. This pattern has also been observed in a heterozygote patient with a “variant phenotype” in filipin staining and with high levels of plasma oxysterols ^60^.

Diffusion tensor imaging (DTI) analyses showed decreased fractional anisotropy in NPC patients compared with controls, especially in the corpus callosum, internal capsule, corona radiate, and the cingulate gyrus, with an early but transient improvement of DTI metrics after miglustat treatment ^61– 63^. Global callosal measures correlated significantly with duration of illness and symptom score and at trend level with degree of filipin staining ^64^.

In agreement with these studies, myelin water imaging, a technique that measures the amount of water present within the myelin of white matter tracts ^65, 35^, has shown large reductions of myelin water fraction in large association tracts and the corpus callosum, paralleling prior reports of reduced callosal fractional anisotropy and cortical thickness of the corpus callosum ^56, 64, 66, 67^.

Functional imaging with ^18^F-fluorodeoxyglucose positron emission tomography (FDG-PET) and SPECT has highlighted the involvement of frontal and temporal structures, even in the very initial phases of the disease ^28, 68– 72^, possibly reflecting disease severity ^68^.

Proton magnetic resonance spectroscopic imaging (H-MRSI) studies have shown a decreased N-acetyl aspartate/creatine ratio in the frontal and parietal cortices, centrum semiovale, and caudate nucleus, and there were significant correlations between clinical staging scale scores and H-MRSI abnormalities ^59, 73, 74^.

As it clearly emerges, MRI is the modality of choice for identifying reported abnormalities in the clinical setting, such as frontal lobe and cerebellar atrophy ^4, 75^, white matter hyperintensities in parieto-occipital periventricular regions ^75^, deep gray matter and hippocampal atrophy particularly in adult-onset patients ^54^, and reduced midbrain-to-pons ratio ^59^. However, specific neuroimaging findings are lacking in NPC, providing little aid in the clinical setting and diagnostic workup, highlighted by the absence of imaging tests in diagnostic criteria or from the NPC suspicion index ^76^. Furthermore, these biomarkers have not been validated regarding accuracy in the differential diagnosis with other neurodegenerative diseases or compared with healthy controls; thus, specificity and sensitivity measures are currently unavailable. However, new imaging modalities have provided a solid basis for the development of biomarkers to understand disease pathophysiology and to monitor disease progression and response to treatments in the research setting.

## Neurophysiology

As patients with NPC can experience any type of seizure (partial/focal, generalized, absence, myoclonic, or tonic-clonic), which vary in intensity and frequency, electroencephalography (EEG) should be used for confirmation and for differentiating epilepsy from cataplexy ^3, 77– 83^. Patients who develop severe epilepsy generally have a worse prognosis and reduced life span compared with patients who are seizure-free ^3^.

Neurophysiological evaluation with evoked potentials has shown the involvement of central tracts in most patients with NPC. Indeed, somatosensory evoked potentials have been shown to be impaired in patients with NPC, particularly in the lower limbs ^84, 85^, whereas brain auditory evoked potentials showed a bilateral absence of most waves, highlighting the involvement of auditory pathways ranging from the auditory nerve to the midbrain ^84– 87^.

Pyramidal involvement ^85, 88^ and abnormalities of visual evoked potentials ^85^ have been reported in patients with infantile-onset NPC but appear only at advanced stages of disease ^84^. Peripheral neuropathy has also been reported as a rare manifestation in patients with NPC ^3, 68, 89, 90^.

Recently, transcranial magnetic stimulation (TMS) paired-pulse paradigms have been used to evaluate cortical excitability and intracortical connectivity measures. In this context, an impairment in short-latency afferent inhibition (SAI), a measurement of sensorimotor integration, has been observed in NPC. SAI is thought to be mediated largely by central cholinergic transmission and has been shown to be impaired in patients with AD ^91, 92^, further supporting the link between NPC and AD ^60, 93, 94^. Moreover, long-term potentiation (LTP)-like cortical plasticity, evaluated with a paired associative stimulation protocol, has been shown to be impaired in patients with NPC and in a symptomatic heterozygous carrier, confirming previous reports of impaired hippocampal synaptic plasticity in *Npc1*-mutant mice ^95^. The impairment in LTP-like cortical plasticity has also been observed in patients with AD and FTD, further highlighting the possible parallelism between these disorders ^96, 97^. Interestingly, after 12 months of treatment with miglustat, a considerable improvement in SAI and LTP-like plasticity was observed in patients with NPC ^60^.

Only a limited decrease in short-interval intracortical inhibition, a marker of GABAAergic transmission, and intracortical facilitation, a marker of glutamatergic transmission, has been observed. Long-interval intracortical inhibition, reflecting GABABergic transmission, was reported to be within normal range ^60^.

In conclusion, neurophysiological tests still fall short in providing invaluable information for the diagnostic workup of NPC diagnosis because findings are not specific for NPC and may not be found in all cases of NPC. EEG remains the exam of choice for confirming and differentiating epilepsy from cataplexy and for monitoring response to antiepileptic drug treatment.

However, in the research setting, a new series of non-invasive tests have shown a selective impairment of specific intracortical circuits that, if confirmed in a wider population of patients, might provide valuable information on disease pathophysiology and disease progression and possibly be used to monitor response to therapeutic interventions.

## Neuropathology

Although cholesterol is able to flow freely through most cellular membranes, it cannot exit from lysosomes without the aid of NPC1 and NPC2 proteins. The so-called “hydrophobic handoff” model has been proposed ^98^, and recent studies with structural biology approaches, such as crystallography and cryo–electron microscopy, have corroborated this model hypothesis, highlighting how the luminal NPC2 protein picks up cholesterol from endocytosed cholesterol as well as from the significant lipid content present in the lumen of degradative lysosomes, eventually interacting with the membrane-bound NPC1 protein ^10– 19^. However, how cholesterol is transferred across the membrane by the NPC1 protein is still a matter of debate ^99^.

Both NPC1 and NPC2 proteins are involved in the trafficking of low-density lipoprotein (LDL)-derived cholesterol from the lysosome to the cellular membranes of the endoplasmic reticulum, Golgi apparatus, and plasma membrane ^17, 100^. The decrease of cholesterol levels in the endoplasmic reticulum consequently enhances the synthesis and uptake of cholesterol by the sterol response element–binding protein pathway, leading to the accumulation of cholesterol and other lipids in many types of cells, including lipid-laden macrophages (called foam cells) and neuronal and glial cells ^101– 103^.

Compared with most other lipid storage disorders, NPC does not arise from defective substrate degradation but from the impairment of LDL-derived cholesterol export out of the lysosome, followed by the disruption of lipid homeostasis ^103^, affecting multiple cellular functions such as lysosomal calcium homeostasis ^104^, oxidative stress ^105, 106^, Rab-mediated vesicle trafficking ^107, 108^, or fusion of lysosomes ^109^, leading to impaired autophagy ^110, 111^. The accumulation of lipids in the central nervous system causes neuronal distension, axonal swelling, and the formation of axonal spheroids ^112– 115^.

NPC is also characterized by the accumulation of β-amyloid ^116, 117^ and neurofibrillary tangles ^24, 118– 122^, which are immunologically and ultrastructurally similar to those seen in AD ^123^. Indeed, in one of the first descriptions of NPC neuropathology, a widespread neurofibrillary degeneration with a distribution similar to that of advanced AD was reported ^124^. However, subsequent studies highlighted a somewhat different distribution of neurofibrillary tangles in NPC compared with AD ^71^; there was a primary involvement of subcortical structures, including hippocampus, thalamus, and striatum in NPC ^24, 118– 120^, and a more cortical distribution in AD ^125– 127^. Neurofibrillary tangles in NPC tend to be associated with lipid accumulations in swelling neurons, possibly suggesting a triggering effect of intracellular accumulations on tau aggregation ^71, 118^.

Beyond cholesterol pathway NPC and AD have other similarities, which involve AD pathogenesis ^128^. This parallelism between NPC and AD is further strengthened by the observation that cholesterol levels may modulate the processing of amyloid precursor protein ^129^ and accumulation of β-amyloid ^130^ is supported by the disease-modifying effect of the ε4 isoform of apolipoprotein E on disease progression in both NPC and AD ^131– 133^. Moreover, a possible effect of mutations in the NPC genes as AD risk factors has been speculated ^133, 134^.

Just recently, a novel link with another intracellular proteinopathy has been established. Indeed, both in NPC mouse and in a human neuronal model of the disease, an altered expression or mislocalization of the TAR-DNA binding protein 43 (TDP-43) or both were reported ^135^. From a functional point of view, the TDP-43 mislocalization observed in human experimental neuronal models of NPC was associated with specific alterations in TDP-43 controlled genes. Most interestingly, N-acetyl-cysteine or 2-hydroxypropyl-β-cyclodextrin may partially restore TDP-43 metabolism ^135^. TDP-43 inclusions have been reported as the main pathological signature of FTD due to *C9orf72* and *GRN* mutations ^136^ and are also described in AD cases ^137^.

Whereas β-amyloid and α-synuclein may accumulate biochemically in the NPC brain, senile plaques or Lewy bodies are not characteristic of the disease, and further studies are needed to assess the possible overlap between these neurodegenerative disorders. As highlighted above, these speculations have not been validated and replicated in larger studies and thus a cautious interpretation is warranted.

## Treatments

Miglustat, a small iminosugar molecule that reversibly inhibits glycosphingolipid synthesis, has been licensed in the European Union and elsewhere for the treatment of progressive neurological manifestations of NPC in both children and adults ^3, 13, 83, 106^. It has been shown to stabilize or improve certain neurological manifestations in six clinical trials ^28, 88, 138– 142^, none of which is randomized or placebo-controlled, and to partially restore neurophysiological markers of cholinergic impairment (such as SAI) and LTP-like cortical plasticity ^60, 95^.

The effect of cholesterol-lowering agents on hepatic and plasma cholesterol in NPC has been assessed with dimethyl sulfoxide, nicotinic acid, lovastatin, cholestyramine, and combinations of the above drugs. The treatment effects on total cholesterol varied depending on the drug combinations and overall improved with the number of drugs. However, efficacy for neurological outcomes was not reported, and safety findings discouraged widespread application of cholesterol-lowering agents to patients with NPC ^143, 144^. Many other therapies, including 2-hydroxypropyl-β-cyclodextrin (NCT02912793, NCT02939547, NCT01747135, and NCT02534844), arimoclomol (NCT02612129), vorinostat (NCT02124083), lithium carbonate (NCT03201627), and δ-tocopherol, are currently under clinical investigation for NPC ^145^.

Although the mechanism of action of 2-hydroxypropyl-β-cyclodextrin is not fully understood, studies in animal models have shown reduced cholesterol and sphingolipid storage and liver function improvement, lower degree of neurodegeneration, and better survival following intravenous, subcutaneous, intracerebroventricular, or intrathecal administration, for both *NPC1* and *NPC2* mutations ^146– 154^.

Just recently, a phase 1–2 clinical trial with monthly 2-hydroxypropyl-β-cyclodextrin was performed on 14 patients with NPC and showed slowed disease progression, in particular in ambulation, cognition, and speech, with an acceptable safety profile ^155^. Since 2-hydroxypropyl-β-cyclodextrin does not efficiently cross the blood-brain barrier ^156^ and high-dose systemic delivery can be associated with pulmonary toxicity ^149, 157^, lumbar intrathecal administration is the route of choice but has common adverse events, such as post–lumbar puncture headache, reported in 64% of cases ^155^. At doses above 600 mg, unexpected adverse events included post-administration unsteadiness and fatigue, which were transient and typically occurred 24 to 72 hours after dosing ^155^. Ototoxicity, with mid- to high-frequency hearing loss, an expected adverse event, was documented in all participants and was probably due to outer hair cell loss ^155, 158^.

The main drawback of this approach is due to the route of administration; owing to the ability of molecular chaperones of the heat shock protein 70 (HSP70) family to protect pathologically challenged cells, HSP70-based therapies are emerging as attractive treatment options for many degenerative diseases ^159– 163^, including lipid storage disorders due to their direct interaction with lysosomes ^162, 164^, and for the proper folding and activity of the NPC1 protein ^165, 166^. In this view, arimoclomol and small-molecule HSP70 co-inducer have been tested in a number of clinical trials ^167, 168^ and are currently under investigation for the treatment of NPC.

Vorinostat, currently used for cutaneous T-cell lymphoma, is a histone deacetylase inhibitor that was able to increase NPC1 protein and decrease unesterified cholesterol deposits ^169– 173^. Indeed, in selected genetic disorders, histone deacetylase inhibitors have been shown to induce histone modifications that not only can result in increased or decreased transcriptional expression of mutated genes ^174^ but also confer indirect benefits through acetylation of non-histone proteins, such as transcription factors and heat shock proteins, that modulate chaperones and proteostatic networks ^169, 174– 176^. Interestingly, however, treatment of *NPC2*-deficient human fibroblasts with a histone deacetylase inhibitor did not reduce cholesterol storage in lysosomes and late endosomes ^171^.

FTY720/fingolimod, an inhibitor of class I histone deacetylases used for the treatment of multiple sclerosis, has been shown to increase the expression of NPC1 and NPC2 in human *NPC1*-mutant fibroblasts and to significantly reduce the accumulation of cholesterol and glycosphingolipids ^177^.

Other therapeutic approaches—including several that use human stem cells, such as hematopoietic stem cell transplantation (NCT00668564, NCT00730314, and NCT01372228), human placental-derived stem cell transplantation (NCT01586455), and intrathecal umbilical cord blood–derived oligodendrocyte-like cells (NCT02254863)—are currently under development for the treatment of NPC.

In regard to *NPC2* mutations, given that the NPC2 protein is soluble, secreted, and recaptured, there is a rationale supporting early hematopoietic stem cell transplantation ^178, 179^.

Other attractive approaches, evaluated in preclinical models of disease, include the systemic delivery of adeno-associated virus vectors to *NPC1* ^-/-^ mice to increase the expression of a therapeutic *NPC1* transgene, which has resulted in an improved clinical appearance, delayed weight loss, significantly increased life span, reduced cholesterol storage, and decreased cerebellar Purkinje cell degeneration compared with untreated *NPC1* ^-/-^ mice ^180, 181^.

## Conclusions

NPC represents an autosomal recessive disorder with extensive biochemical, molecular, and clinical variability, which probably results in an underestimation of the burden of NPC cases worldwide. The relatively low incidence of the disease increases the difficulties in developing high-quality observational studies or randomized clinical trials.

As outlined above, recent studies have tried to shed light on the pathophysiology of this disease, further underlying its complex nature. Nevertheless, numerous biomarkers reflecting disease pathogenesis have emerged, thus representing a useful aid to diagnose disease or to evaluate disease progression and response to therapeutic interventions. Indeed, in this view, imaging and neurophysiological markers have been shown to reflect disease severity and to respond to disease-modifying treatments.

Intriguingly, a close parallelism has been observed between NPC and other neurodegenerative disorders, highlighting the possible involvement of multiple, but intertwined, disease pathways. Thus, unravelling the connection between neurofibrillary tangles, TDP-43 pathology, and neurodegeneration could result in important advances not only for NPC but also for AD and FTD/amyotrophic lateral sclerosis.

Diagnosing NPC represents a challenge for physicians, and delays in diagnosis and ensuing miglustat treatment and eventually future disease-specific interventions may affect disease outcomes because of irreversible anatomical damage and progressive neurodegeneration ^182^. In this view, a prompt diagnosis is essential, and development of clinical tools, such as suspicion index to provide a risk prediction score ^76^, along with instrumental diagnostic and prognostic markers, is mandatory. Efforts to increase awareness of NPC among clinicians are still needed, but the recent development of rapid and relatively simple instrumental and laboratory tests should improve the diagnostic and prognostic approach to NPC ^49^.
